# Tomato Root Colonization by Exogenously Inoculated Arbuscular Mycorrhizal Fungi Induces Resistance against Root-Knot Nematodes in a Dose-Dependent Manner

**DOI:** 10.3390/ijms23168920

**Published:** 2022-08-10

**Authors:** Sergio Molinari, Masoud Akbarimotlagh, Paola Leonetti

**Affiliations:** 1 Department of Biology, Agricultural and Food Sciences, Institute for Sustainable Plant Protection, Bari Unit, CNR, 70126 Bari, Italy; 2 Department of Plant Pathology, Faculty of Agriculture, Tarbiat Modares University, Tehran 14115-111, Iran

**Keywords:** arbuscular-mycorrhizal fungi, AMF colonization-dependent genes, BCA commercial formulates, *GPX* gene, mycorrhiza-induced resistance, *PR4b* gene, root-knot nematodes, tomato

## Abstract

Arbuscular mycorrhizal fungi (AMF) are generally recognized to induce plant growth and prime plants against soil-borne parasites, such as plant parasitic nematodes. However, the effectiveness of commercial formulates containing AMF has been questioned. Increasing amounts per plant of one commercial AMF-containing formulate, reported in the text as Myco, were used to detect the effects on growth of tomato plants and the resistance induced against root-knot nematodes (RKNs) The doses used per plant (0.5, 1.0, 2.0 g, reported as Myco1, Myco2, Myco3, respectively) were soil-drenched to growing potted plants; the effects of such treatments were analyzed both in plants not inoculated or inoculated by *Meloidogyne incognita* juveniles. Consistent increases in plant weight were apparent as soon as 7 days only after Myco2 treatments. Moreover, only treatments with Myco2 induced a consistent repression of the nematode infection observed in untreated plants. Conversely, treatments with Myco1 and Myco3 did not produce such an early growth improvement; some plant weight increase was observable only at 28 dpt. Accordingly, such Myco doses did not restrict the level of infestation observed in untreated plants. Control of infection was dependent on the dose of Myco provided to plants five days before nematode inoculation. About one month after all Myco treatments, several areas of roots were found to be colonized by AMF, although in Myco2-treated plants, three genes involved in the AMF colonization process (*SlCCaMK*, *SlLYK9*, and *SlLYK13*) were found to be over-expressed already at 7 dpt; over-expression was generally less consistent at 14 and 21 dpt. The expressions of two key genes of plant defense, the hypersensitive cell death inducer *PR4b* gene and the glutathione peroxidase-encoding *GPX* gene, were monitored in roots of Myco2-treated plants 3 and 7 days after nematode inoculation. *PR4b* was over-expressed and *GPX* was silenced in treated plants with respect to untreated plants. The repressive effect of Myco2 treatment against RKN infection was completely abolished when Myco2 suspensions were autoclaved to sterilization or treated with the potent anti-fungal agent amphotericin B, thus indicating that the biological control agents contained in the commercial formulate were living fungi.

## 1. Introduction

Root-knot nematodes (RKNs) are obligate soil-borne animal parasites that cause significant damages to most crops worldwide. The invading motile worm-like juveniles (J2s) penetrate the roots and move intercellularly through the cortex toward the central cylinder. They have a protrusible stylet in their mouth apparatus by which they suck cell sap from few cells of the central cylinder and inject an array of digestive compounds secreted by pharyngeal glands. These compounds include effector molecules that, when recognized by intracellular NLR proteins (nucleotide binding domain, NDB, leucine-rich repeats, LRR), induce in plants the so-called effector-triggered immunity (ETI) [1]. Recognition occurs in incompatible resistance gene-carrying plants. The most studied resistance gene present in most commercial resistant tomato cultivars is *Mi-1.2*, which works against the three most diffused RKN species: *Meloidogyne incognita*, *M. javanica*, and *M. arenaria* [2]. ETI of resistant tomato against RKNs is commonly associated with an early hypersensitive reaction (HR) that consists of a prolonged oxidative burst of reactive oxygen species (ROS). The uncontrolled generation of ROS leads to cell death and necrosis of tissues surrounding nematode head, which forces the invading juvenile (J2) to starve or leave the root. In susceptible tomato plants, injected or secreted effectors are not recognized, do not trigger plant immunity, and act as suppressors of plant defense, thus leading to silencing or down-regulation of many defense genes [3,4]. Therefore, J2s soon become sedentary and, through two molts as J3 and J4, develop into adult gravid females, which lay 200–400 eggs in gelatinous egg masses outside the roots. Moreover, nematode secretions produce hypertrophy and hyperplasia of the root tissue that appear as the familiar visible galls or knots. Susceptible plants can be primed or immunized against RKN infection by some chemical activators, such as salicylic acid (SA) and its functional homologues, benzol-(1,2,3)-thiadiazole-7-carbothionic acid *S*-methyl ester (BTH) and 2,6-dichloroisonicotinic acid (INA) [5,6]. Exogenously provided SA has been proved to induce systemic acquired resistance (SAR) in tomato against RKNs [7].

Moreover, symbiosis of arbuscular mycorrhizal fungi (AMF) with roots of most plants produces a mycorrhiza-induced resistance (MIR), acting against numerous different pathogens, such as root nematodes and chewing insects and necrotrophic pathogens and generalist chewing insects in above-ground tissues [8]. AMF belong to Glomeromycota and establish an intimate relationship by entering root cells and developing specialized structures into the cytosol known as arbuscules [9]. The ability of AMF pre-colonization to induce plant resistance to subsequent RKN infection has been extensively reported [4,10,11,12]. However, most studies use laboratory-selected or naturally collected strains of AMF [11,12]. Presently, AMF are being considered and used as “biofertilizers”, although their use is limited by difficulties in the production process [13]. The use of commercial products can help standardize treatments and produce for farmers’ protocols for each cropping system. Therefore, we focused our investigation on AMF-containing commercial formulates to pave the way for practical application and utilization of AMF as plant immunity activators and biocontrol agents in conventional and organic agriculture [4]. The practical use of commercial AMF formulates as biocontrol agents is presently not so diffused yet because the many conditions needed to make them properly work in the various cropping systems are still poorly investigated and applied. Most past failures, then, have probably been consequent to unsuitable treatments. Indeed, successful performances depend on an array of factors, and until we elucidate the functional variations that make root colonization effective against pests, practical applications of AMF, contained or not in commercial formulates, will not move forward. The growth stage of plants treated with biological control agents has recently been found to be of paramount importance to determine the performance against RKNs [14]. In this study, we used three different doses of an AMF-containing commercial formulate (reported as Myco) to test the performance against RKNs when they were soil-drenched to potted tomato young plants. Data are reported to show that roots of treated plants are actually colonized by AMF, although such a colonization may be effective against RKN attack only when determined amounts of formulate per plant are used. Finally, expressions of genes involved in AMF colonization and plant immunity were evaluated in roots of plants treated with the most effective Myco dose against nematodes.

## 2. Results

### 2.1. Colonization of Exogenously Added AMF to Tomato Roots

**Figure 1:**
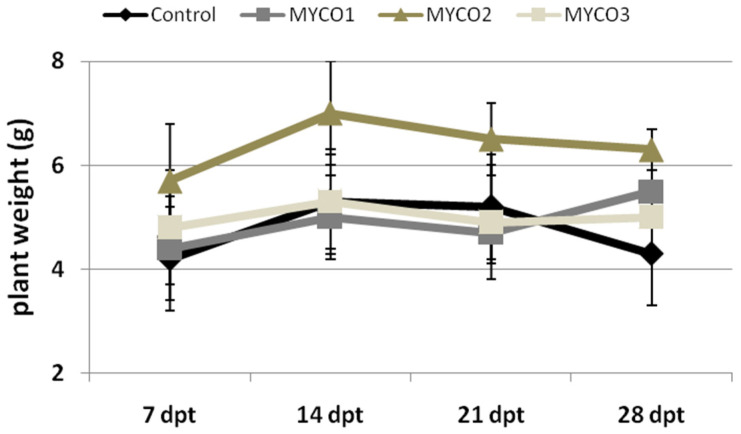
Weights of tomato plants untreated (Control) and treated with 3 different doses of a formulate containing AMF (Myco1, Myco2, Myco3), at 7, 14, 21, and 28 days after treatment.

Tomato plants were soil-drenched with suspensions of a commercial formulate (Myco) containing AMF in different amounts expressed as g Myco per plant: (i) 0.5 (Myco1); (ii) 1.0 (Myco2); (iii) 2.0 (Myco3). Plant growth, expressed as plant weight, was monitored from 7 to 28 days post-treatment (dpt) and compared with that of growing control untreated plants (Figure 1). Plants provided with Myco2 appeared to enhance their weight compared with plants subjected to treatments with different Myco doses over the entire experimental period. Growth of plants treated with Myco1 and Myco3 did not differ with that of control plants, except at 28 dpt, when treated plants showed higher weights than controls.

**Figure 2:**
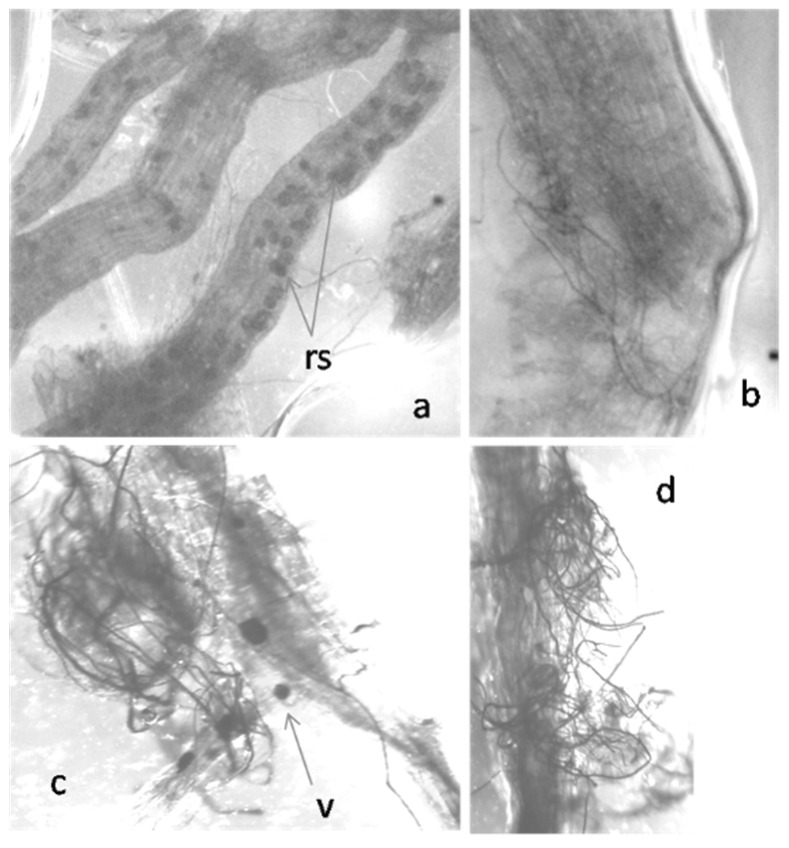
Tomato roots infected with arbuscular mycorrhizal fungi, cleared and stained with KOH-lactophenol blue treatment. (**a**) Roots invaded by resting spores (rs) × 50; (**b**) intra-radical hyphae × 50; (**c**) AMF-infected area with a visible vesicle (v) × 100; (**d**) an extensively AMF-infected area × 100.

At 28 dpt, we checked if higher growth rate of treated plants was determined by AMF colonization of roots. At that time after treatment, roots of plants soil-drenched with all the tested doses were found infected by AMF, as shown in Figure 2.

**Figure 3:**
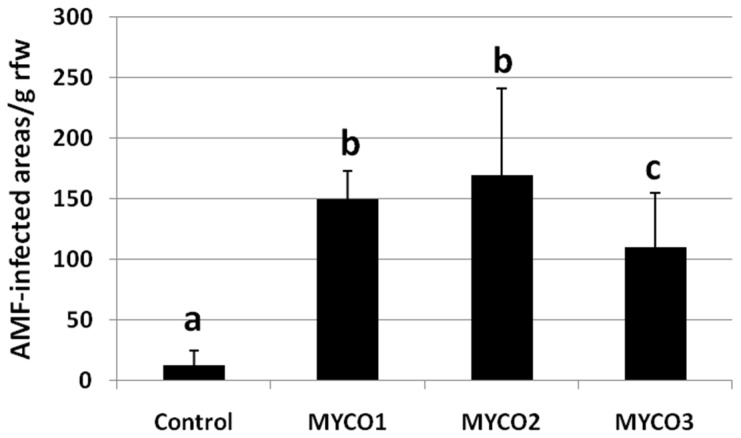
AMF-infected areas per g of root fresh weight (rfw) observed on KOH-lactophenol blue treated roots under a stereoscope. Roots were collected from plants untreated (Control) or treated with 3 doses of Myco at 28 dpt. Six different root samples were analyzed per treatment. Values are expressed as mean (*n* = 6) ± standard deviation. Means were separated by Duncan’s test; different letters indicate significantly different means (significance level: 0.05).

Numbers of AMF-infected areas per root weight, detected at 28 dpt, revealed a diffused infection in plants treated with Myco1 and Myco2, with respect to Myco3; untreated plants showed negligible infection (Figure 3).

**Figure 4:**
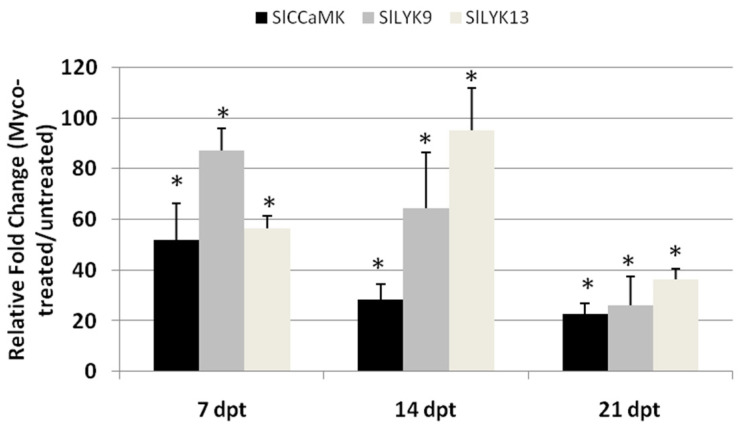
Expression of *SlCCaMK*, *SlLYK9*, and *SlLYK13* genes in tomato roots after treatment of plants with Myco2. Expression of genes was detected by quantitative real-time reverse-transcription polymerase chain reaction (qRT-PCR) in roots of tomato plants at 7, 14, and 21 days post-treatment (dpt). Data are the mean fold changes (*n* = 6) ± SD in gene transcript levels of roots from Myco2-treated plants relative to those from untreated control plants (the value 1 indicates no change). An asterisk (*) indicates that the mean fold change is significantly different from 1 as determined by the non-parametric Kolmogorov–Smirnov test (*p* < 0.05).

To confirm the establishment of AMF–tomato symbiosis in roots treated with an AMF-containing commercial formulate, the relative expression of three genes, calcium- and calmodulin-dependent protein kinase (*SlCCaMK*) and lysin motif receptor-like kinase 9 and 13 (*SlLYK9*, *SlLYK13*), previously reported to be involved in AMF colonization to tomato roots [15], were detected in roots of Myco2-treated plants compared with untreated plants at 7, 14, and 21 dpt (Figure 4).

**Figure 5:**
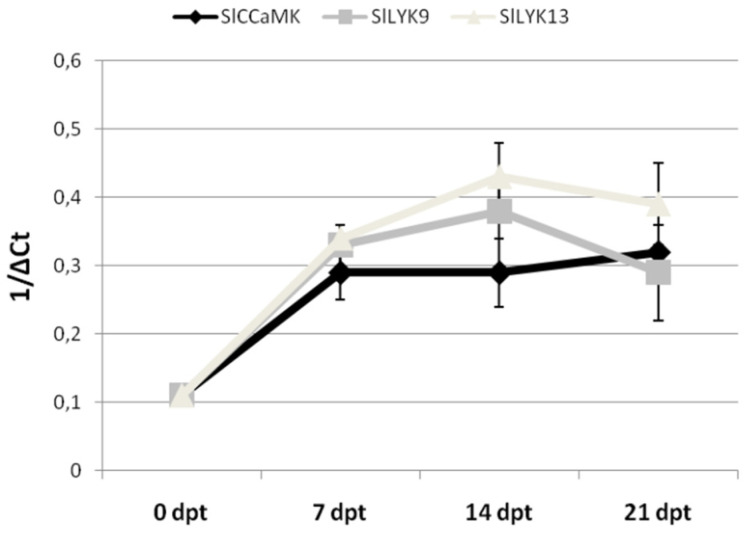
Expression of *SlCCaMK*, *SlLYK9*, and *SlLYK13* genes in tomato roots after treatment of plants with Myco2. Expression of genes was detected by quantitative real-time reverse-transcription polymerase chain reaction (qRT-PCR) in roots of tomato plants at 0, 7, 14, and 21 days post-treatment (dpt). Gene transcript levels are expressed as 1/ΔC_t_, where ΔC_t_ is the difference between the cycle thresholds of fluorescence signal of the tested gene and the signal of the reference gene (*Actin 2*). Data are mean (*n* = 6) ± SD.

In the first 14 days from Myco2 treatment, expression of all the tested genes in roots was 50–100-fold higher than that from roots of untreated plants. *SlCCaMK* and *SlLYK9* gene expression was the highest at 7 dpt and then decreased with time, whereas the highest *SlLYK13* gene expression was at 14 dpt. The increase in gene expression indicates that the rate of AMF colonization of roots mostly occurred in the first 7 days after treatment; in the subsequent week, gene expression showed a slight or no increase at all with respect to the level reached at the first week. At the end of the third week, the gene expression rate was again similar to that registered at the end of the first week (Figure 5).

### 2.2. Myco Treatments to Tomato Roots Induces Resistance to RKNs in a Dose-Dependent Manner

**Figure 6:**
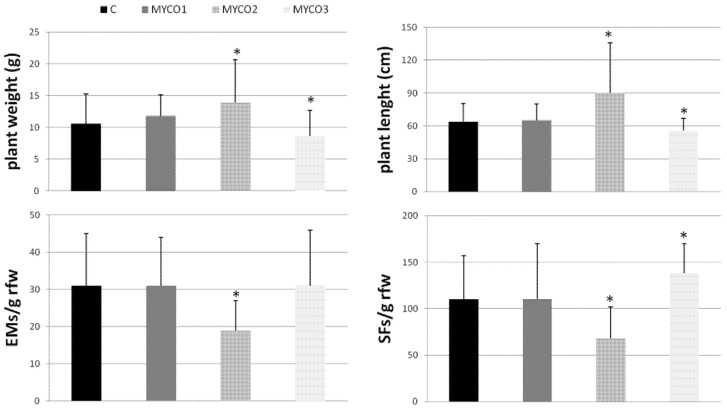
Plant growth and nematode infection factors of tomato plants untreated (C) and treated with 0.5, 1.0, and 2.0 g Myco/plant (MYCO1, MYCO2, and MYCO3, respectively). Plants were inoculated with RKN J2s and harvested after 40 days. Plant growth was evaluated by plant weight and length; nematode infection by the numbers of egg masses (EMs) and sedentary forms (SFs) per gram of root fresh weight (g rfw). Values are expressed as mean (*n* = 12) ± SD; means of untreated plants factors were separated from those of Myco-treated plants by a *t*-test (* *p* < 0.05).

Untreated and Myco-treated tomato plants were inoculated with active J2s of the RKN *M. incognita*. At the end of nematode life cycle, plant growth and the level of infection were evaluated. Control plants and plants treated with the lowest Myco dose tested (Myco1, 0.5 g Myco/plant) showed very similar rates of growth and infection indices. Plants treated with Myco2 (1 g Myco/plant) had significant increases in plant growth parameters and almost halved infection severity. Plants treated with Myco3 (2 g Myco/plant) had an even higher number of developing individuals in roots (SFs) than controls; more galled and damaged roots led Myco3-treated plants to a reduced growth with respect to untreated plants (Figure 6).

### 2.3. Resistance Induction against RKNs Is Mediated by AMF Colonization

Since colonization of roots by the AMF contained in the used formulate had been proven, experiments were arranged to bring evidence of an association of AMF colonization with the observed restriction of nematode infection. Myco2 suspensions were either autoclaved to sterilization or added with amphotericin B, a potent anti-fungal agent; such altered suspensions were provided to plants and their effects on plant growth and nematode infection were compared with those induced by unaltered suspensions (Table 1).

Sterilization by autoclavation and addition of the anti-fungal compound amphotericin B in Myco2 suspensions completely abolished the restriction of nematode infection caused by unaltered Myco2 suspensions. Surprisingly, control plants provided with sterilized water and amphotericin B solutions showed numbers of SFs/g rfw significantly lower than plants provided with distilled water.

### 2.4. AMF-Mediated Plant Immunity against RKNs

**Figure 7:**
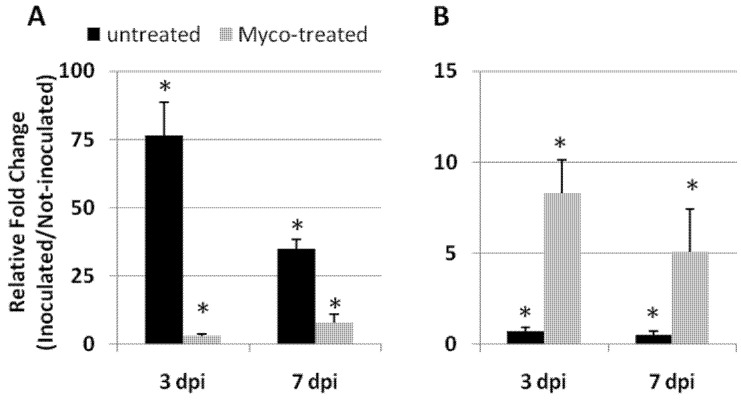
Expression of *GPX* (**A**) and *PR-4b* (**B**) genes in tomato roots of untreated and Myco-treated plants by quantitative real-time reverse-transcription polymerase chain reaction (qRT-PCR). Relative fold changes (the value 1 indicates no change) of nematode inoculated with respect to not inoculated plants were detected at 3 and 7 days post-inoculation (dpi). Data are the mean fold changes (*n* = 6) ± SD in gene transcript levels. An asterisk (*) indicates that the mean fold change is significantly different from 1 as determined by the non-parametric Kolmogorov–Smirnov test (*p* < 0.05).

Immune response of plants to incompatible pests relies on ROS augmented generation and secretion of a series of defense proteins, including pathogenesis-related (PR) proteins [16]. Successful nematode infections imply a potentiation of ROS scavenging systems, whereas successful plant immune response is associated with their inhibition [4]. The expressions of the glutathione peroxidase encoding gene (*GPX*), which is considered as a potential detoxifier of H_2_O_2_ [17], and of the hypersensitive cell death inducer *PR4b* gene [18] were monitored 3 and 7 days post-inoculation (dpi) in roots of Myco-treated plants and compared with those of untreated plants (Figure 7); *GPX* and *PR4b* gene expressions were detected to have evidence of induced immunity against RKNs conferred by AMF colonization of roots.

*GPX* expression was highly enhanced in compatible nematode–tomato interaction in the first week after inoculation; on the contrary, pre-treated primed plants by AMF had their *GPX* expression notably reduced. When plants were pre-treated with Myco, immunity against RKNs was promoted by AMF root colonization, as indicated by the marked increase in the expression of *PR-4b*, which mediates hypersensitive cell death. Conversely, nematode-inoculated susceptible plants had consistently reduced levels of *PR-4b* transcripts with respect to not inoculated plants when not previously treated with Myco.

## 3. Discussion

Most natural field soils contain indigenous mycorrhizal fungi, although nowadays, in most agricultural areas, indigenous mycorrhizal populations have been eradicated or drastically reduced by human influence or natural disturbances. Moreover, when indigenous mycorrhizal fungi may be present in crop areas, their ability to benefit plant growth or to limit pest infections would be influenced by their colonization capacity, that is, the amount of root colonized in a certain time, or the time taken to colonize part of roots by a determined dose of inoculum [19]. If indigenous fungi have a low colonization capacity, it is necessary to turn to commercial formulates containing effective AMF that may increase plant growth and pest resistance. However, there is an array of conditions by which exogenously inoculated AMF may or may not be effective on a determined crop [20]. One of the most important of these conditions has recently been found to be a suitable ratio between the inoculum dose and plant development stage [14]. In this study, an AMF-containing commercial formulate was provided to tomato plants in three increasing doses, which determined the colonization capacity of the inoculated mycorrhizal fungi. In terms of both growth and resistance to RKN infection, plants treated with the extreme doses (Myco1, Myco3) did not differ from plants that were left untreated as controls; rather, Myco3-treated plants were more exposed to nematode damage. Conversely, the medium dose (Myco2) induced a consistent increase in plant weight after treatment, and a lower infection if pre-treated plants were inoculated with nematodes. If growth enhancement is considered as the result of successful AMF colonization of roots, it can be postulated that such a colonization occurs as soon as 7 dpt in Myco2-treated plants and that it is delayed when plants are treated with different doses. Comparably, genes associated with AMF colonization were over-expressed in roots of Myco2-treated plants already at 7 dpt. Nutrient exchange between AMF and roots takes place in the cells surrounding arbuscules, established by a common symbiosis signaling pathway (CSSP) in which the calcium- and calmodulin-dependent protein kinase (CCaMK) plays an important role. CCaMK and the lysin motif receptor-like kinase family (LysM-RLK) are both strictly involved in the establishment of AM [15]. We detected the expression rate of the genes encoding such proteins by qRT-PCR in roots from plants untreated or treated with the most effective of Myco doses (Myco2), until 21 dpt. Expression rates of SlCCaMK and SlLYK9 in roots were found to be about 50–80-fold higher in treated compared with untreated plants. Indeed, the highest rate of gene transcription activity was reached in the first week after AMF inoculation. Therefore, it is likely that the AMF amount provided to plants with Myco2 treatments had a higher colonization capacity than that of different treatments; higher colonization capacity probably resulted in a faster diffused colonization process that allowed plants to be primed against nematodes before inoculation, which was carried out 5 days after treatments. Therefore, effectiveness of exogenously inoculated AMF in resistance induction to RKNs may depend on either suitable doses or suitable time to allow fungi to establish symbiosis with roots. These findings confirm that treatments with immune system activators must be preventive to pest attack. Curative interventions, when infection is settled, may be not effective.

The ability of plants treated with AMF to suppress RKN infection has long and extensively been reported [4,11,21]. This suppressive effect is most likely exerted through an induced systemic resistance (ISR) that, in the case of AMF, has been named MIR [8]. Although activation of plant immune response and enhanced expression of PR-genes have recently been ascertained [4], other mechanisms may be involved in the limitation of nematode infection by AMF-colonized roots, such as higher nutrient uptake, direct competition for nutrients and space, and altered rhizosphere interactions [12]. Interestingly, in this study, MIR against RKNs was found to be associated with the synthesis of the cell death-inducing pathogenesis-related protein PR-4b and with a consistent reduction in the expression rate of GPX, the gene encoding for the most active anti-oxidant enzyme glutathione peroxidase; conversely, GPX expression was highly induced when nematode attacked susceptible not AMF-primed plants.

Although root colonization by AMF and MIR were evident in plants treated with Myco2, we wanted to ascertain if nematode infection restriction was due exclusively to the beneficial fungi contained in the tested commercial formulate. Myco2 suspensions were then incubated with a fungal growth inhibitor (amphotericin B) or autoclaved to sterilization. Restrictions of nematode infection were totally abolished by using these modified Myco2 suspensions, thus proving that induced resistance to RKNs relied on living fungi.

## 4. Materials and Methods

### 4.1. Treatment of Plants with Arbuscular Mycorrhizal Fungi

Seeds of the tomato (*Solanum lycopersicum* L.) cultivar Roma VF, susceptible to root-knot nematodes (RKNs), were surface-sterilized by immersion in 20% bleach for 10 min in vacuum, and sown in a sterilized mixture of peat and soil at 23–25 °C in a glasshouse. Seedlings were transplanted to 110 cm^3^ clay pots filled with a freshly field-collected loamy soil. Pots were put in temperature-controlled benches (soil temperature 23–25 °C) located in a glasshouse, provided with a regular regime of 12 h light/day, and fertilized weekly with Hoagland’s solution [22]. Plants were allowed to grow to a weight of 4–5 g. Colonization by arbuscular mycorrhiza forming fungi (AMF) was allowed by soil-drenching plants with a commercial formulate (Micosat F^®^, named Myco in the text, C.C.S. Aosta, Italy) consisting of 40% root fragments containing intraradical spores/vesicles and hyphae of *Glomus* spp. (*Glomus* spp. GB 67. *G. mosseae* GP11, *G. viscosum* GC 41). The commercial formulate also contained rhizobacteria and yeasts. Three different doses were tested, expressed as g Myco g^−1^ plant fresh weight (pfw): (i) 0.5 (Myco1); (ii) 1.0 (Myco2); (iii) 2.0 (Myco3). Amounts of formulate were dissolved in sterile distilled water and incubated in an orbital shaker at 25 °C for 3 days in dark. Myco2 was the only dose that was able to restrict RKN infection. Therefore, in some experiments, suspensions of Myco2, and the corresponding controls, were either added with 100 µg mL^−1^ amphotericin B, a potent antifungal compound, to exclude the fungal component from the soil-drenched formulate, or autoclaved at 121 °C for 30 min to exclude living microorganisms.

Fresh weights in grams of plants untreated and treated with different doses of Myco were measured at 7, 14, 21, and 28 days after treatment (dpt).

### 4.2. Root Colonization

Roots untreated and treated with different doses of Myco were collected 28 dpt and stained to detect AMF colonization by the method of lactophenol blue [23]. Fresh roots were gently washed with water to remove attached soil particles and soil debris. Then, they were chopped in short pieces and immersed in 10% KOH for 45 min at 90 °C in a water bath located under a fume hood. Roots were thoroughly rinsed with water and acidified in 1% HCl. Staining was performed by soaking the roots in 0.05% lactophenol blue in a 90 °C water bath for 30 min and destained in 30% methanol/10% acetic acid distilled water solution. Destained roots were stored in a refrigerator before being observed under a dissecting microscope (Leica M 125) equipped with a LEICA IC80 HD photo camera (Leica Geosystem, Germany). Discrete, blue-stained areas containing typical fungal structure (i.e., hyphae, vesicles/spores, and arbuscules) were observed and counted per gram fresh weight of main root to express the intensity of root colonization.

### 4.3. Nematode Inoculations and Infection Level Determination

One population of the root-knot nematode *Meloidogyne incognita* (Kofi *et* White) Chitw., collected in one eggplant field in Italy and reared on susceptible tomato in a glasshouse, was used for inoculation of Myco-treated and untreated plants. Active second-stage juveniles (J2s) were obtained by incubation of egg masses in tap water at 25 °C. At the third day of incubation, J2s were collected and concentrated by filtering through 500 mesh sieves. Amounts of J2s mL^−1^ were measured under a dissecting microscope at 25× magnification. Plants in pots were inoculated with 300 J2s each by pouring suitable volumes of a stirring J2 suspension into 2 holes made in the soil at the base of plants. Inoculation was carried out 5 days after Myco treatment.

Under the adopted experimental conditions, nematodes completed their life cycle with the deposition of eggs outside the roots in gelatinous masses in about one month. Afterwards, J2s start to hatch in the pot soil and re-infest roots as a second generation. Secondary infection is performed by a number of J2s much higher than that exogenously applied for the first one. Plants were harvested 40–60 days after inoculation to let second-generation J2s enter the roots and develop into sedentary forms (J3, J4, swollen females) but not to reproductive egg-laying females. Therefore, production of egg masses (EMs) is an index of the severity of J2s artificial infection, whereas the number of sedentary forms (SFs) developed into the roots is primarily an index of the heavier secondary infection. Moreover, EM numbers indicate the reproductive rate of the nematode population performed on untreated or Myco-treated plants, whereas SF numbers reflect the galling state and damage level of the roots.

At harvest, plants were weighed and their length measured. Then, roots were cut from shoots and washed free of soil debris. Roots were chopped into fragments; groups of these fragments were weighed and used as samples for the determination of the above-mentioned infection parameters, which were expressed as EMs or SFs g^−1^ rfw. Gelatinous masses were red-colored by immersion of root samples in 0.1 g L^−1^ Eosin Yellow and stored in a refrigerator for at least 1 h. Samples were scored for red-colored egg masses under a stereoscope (6× magnification). SF extraction from roots was preceded by an incubation in a mixture of the enzymes pectinase and cellulase at 37 °C in an orbital shaker to loosen the bindings between sedentary nematodes and roots. Afterwards, roots were ground in physiological solution (0.9% NaCl) and the sedentary forms collected on a 90 µm sieve. Aliquots (2 mL) of stirring nematode suspensions were pipetted in small Petri dishes and the number of SFs was counted under a stereoscope (12× magnification).

### 4.4. RNA Extraction, cDNA Synthesis, and Quantitative Real-Time Polymerase Chain Reaction

Roots from untreated and Myco2-treated plants were collected at 7, 14, and 21 dpt. Roots from untreated and Myco2-treated plants inoculated with nematodes were collected at 3 and 7 dpi. Root samples were weighed and immediately used for RNA extraction or stored at −80 °C. Samples were separately ground to a fine powder in a porcelain mortar in liquid nitrogen. An aliquot of macerated tissue (100 mg) per sample was used for RNA extraction. Extractions of total RNA were carried out using an RNA-easy Plant Mini Kit (Qiagen, Düsseldorf, Germany), according to the instructions specified by the manufacturer. RNA quality was verified by electrophoresis runs on 1.0% agarose gel and quantified using a nano-drop spectrophotometer. QuantiTect Reverse Transcription Kit (Qiagen, Germany) with random hexamers was used for cDNA synthesis from 1 μg of total RNA, according to the manufacturer’s instructions. PCR mixtures (20 μL final volume) contained RNAse-free water, 0.2 μM each of forward and reverse primers, 1.5 μL cDNA template, and 10 μL SYBR^®^ Select Master Mix (Applied Biosystems, Monza, Italy). PCR cycling consisted of an initial denaturation step at 95 °C (10 min) and 40 cycles at 95 °C (30 s), 58 °C (30 s), and 72 °C (30 s), with a final extension step at 60 °C (1 min). qRT-PCRs were performed in triplicate using an Applied Biosystems^®^ StepOne™ instrument. The following tomato genes were tested: *calcium- and calmodulin-dependent protein kinase* (XM_004230010.4, *SlCCaMK*), *lysin motif receptor-like kinase 9 and lysin motif receptor-like kinase 13* (*SlLYK9* XM_004247586.4; *SlLYK13* NM_001247801.1), *glutathione peroxidase* (XM_004244468.3, *GPX*), and *pathogenesis-related gene 4b* (NM_001247154.1, *PR-4b*). For each oligonucleotide set, a no-template water control was used. *Actin-7* (NM_001308447.1, *ACT-7*) was used as the reference gene for quantification, as it was tested to be the most suitable one for the experimental conditions used in this work.

The oligonucleotide primers for each gene are listed in Table 2.

The threshold cycle numbers (C_t_) for each transcript quantification were examined and the relative fold changes in gene expression between Myco-treated and untreated roots, or uninfected and uninfected roots, were calculated by the 2^−∆∆CT^ method [24]. Some gene expression data were expressed as 1/ΔC_t_ when comparisons were made on the same set of data over time.

### 4.5. Experimental Design and Statistical Analysis

Experiments to test AMF colonization of roots and its growth promoting effect were designed to use 24 plants for each of the 4 tested treatments. Measurements of weight were taken on untreated and Myco-treated plants 4 times over a 28-day experimental period. Six replications per time point were arranged; values of plant weight in grams are expressed as mean (*n* = 6) ± standard deviation. Numbers of AMF-infected root areas were expressed on a fresh weight unit base. Values are the mean of 6 different measurements at the stereoscope ± standard deviation; means belonging to 4 treatments were separated by Duncan’s test (significance level: 0.05) carried out by X-Stat Basic software. Three different experiments were performed using Myco as a resistance activator to nematode infection; in each experiment, 6 treated and 6 control untreated inoculated plants were used. Means of plant growth and infection factors are the results of 18 replicates, respectively. Mean ± standard deviation of control and treated plants were separated by a paired *t*-test (significance level: 0.05) using Microsoft Excel Software (Microsoft Office LTSC Professional Plus 2021). Three experiments were performed to evaluate the effects of amphotericin B and autoclavation on nematode infection. Twelve replicates were used to obtain the means which were expressed ± standard deviation and separated by Duncan’s test (significance level: 0.05) carried out by X-Stat software.

For RNA extraction, plants coming from 2 independent bioassays were used. Roots from 2 plants of the same bunch constituted one sample. RNA was extracted from 3 different samples of roots per treatment, harvested at each dpt and dpi. qRT-PCR data are expressed as mean (*n* = 6) ± standard deviation of 2^−∆∆Ct^ or 1/ΔC_t_ values of each group from Myco-treated/inoculated plants, considering as 1 the values of each group from untreated/not inoculated plants, taken as controls; significant difference with respect to controls was determined by the non-parametric Kolmogorov–Smirnov test (* *p* < 0.05).

## 5. Conclusions

The data presented herein confirm the suitability of AMF-containing commercial formulate use as a control method against RKNs. However, AMF colonization of roots must be established before nematode attack to ensure primed plants trigger an effective immune response able to limit damages caused by infection. Therefore, optimal doses of the formulates, plant age, time between AMF inoculation, and exposure to nematodes, soil type, cropping conditions, etc., should be investigated and standardized to make AMF treatment a successful and diffused component of integrated pest management in conventional and organic agriculture.

## Figures and Tables

**Table 1:** Plant growth and nematode infection factors of tomato plants treated with (i) sterile distilled water (Cntr) and Myco2 suspensions (MY); (ii) autoclaved distilled water and Myco2 suspensions MYAut; (iii) sterile distilled water and Myco2 suspensions added with 100 µg mL^−1^ amphotericin B (MYAMPHB). Plants were inoculated with RKN J2s and harvested after 60 days. Plant growth was evaluated by plant weight (g); nematode infection by the numbers of egg masses (EMs) and sedentary forms (SFs) per gram of root fresh weight (g rfw). Values are expressed as mean (*n* = 12) ± SD; means of each parameter were separated by Duncan’s test, different letters indicate significantly different means (significance level: 0.05).

	Plant Weight (g)		EMs/g rfw		SFs/g rfw	
	Cntr	Myco2	Cntr	Myco2	Cntr	Myco2
**MY**	15.4 ± 3.9 ^ab^	18.2 ± 2.5 ^a^	52 ± 15 ^a^	32 ± 10 ^b^	277 ± 81 ^a^	173 ± 28 ^c^
**MYAut**	16.4 ± 3.1 ^ab^	17.3 ± 4.4 ^ab^	58 ± 11 ^a^	66 ± 22 ^a^	173 ± 73 ^c^	214 ± 88 ^bc^
**MYAMPHB**	14.4 ± 3.2 ^b^	14.6 ± 3.1 ^b^	51 ± 20 ^a^	55 ± 22 ^a^	189 ± 48 ^bc^	243 ± 78 ^ab^

**Table 2:** Tomato AMF colonization- and defense-related genes examined in this study and the specific primers used in quantitative reverse transcriptase-polymerase chain reaction (qRT-PCR).

Gene Acronym	Primer Sequence (5′-3′)
*SlCCaMK*	F: CATGGGTGAGGGGAGAGTTA R: CATAGCTGCTGCACGAAACTT
*SlLYK9*	F: TGAGCAATTCAATCCATGTCA R: ATGGGGATAAAAGGGGTTG
*SlLYK13*	F: ACTTCTTCTCAAATTGCACAACA R: AAGGGAGATTCGATTCGGGC
*SlGPX*	F: GTTTGCTTGCACACGGTTTA R: CGTCGTTGGTGGATACCTCT
*SlPR-4b/P2*	F: TGACCAACACAGGAACAGGA R: GCCCAATCCATTAGTGTCCA
*ACT-7*	F: CAGCAGATGTGGATCTCAAA R: CTGTGGACAATGGAAGGAC

## Data Availability

Not applicable.
